# Nurses’ steps, distance traveled, and perceived physical demands in a three-shift schedule

**DOI:** 10.1186/s12960-022-00768-3

**Published:** 2022-10-08

**Authors:** Hyoung Eun Chang, Sung-Hyun Cho

**Affiliations:** 1 Research Institute of Nursing Science, College of Nursing, Jeonbuk National University, #712, 567 Baekje-Daero, Deokjin-Gu, Jeonju-Si, Jeollabuk-Do 54896 Republic of Korea; 2 Research Institute of Nursing Science, College of Nursing, Seoul National University, Seoul, Republic of Korea

**Keywords:** Activity tracker, Distance, Nurse, Physical demands, Shift work, Smart band, Steps

## Abstract

**Background:**

The physical job demands of hospital nurses are known to be very high. Although many studies have measured the physical activities of nurses subjectively using questionnaires, it remains necessary to quantify and measure nurses’ physical activity at work using objective indicators. This study was conducted to address this gap in the literature by analyzing nurses’ physical activity using both objective measurements and subjective perceptions. The number of steps, distance traveled, and actual work hours were measured during work, and the influence of related factors was analyzed.

**Methods:**

Using a cross-sectional design, survey and activity tracking data were collected from nurses who worked in three shifts in two tertiary hospitals located in the capital region of South Korea. The participants comprised 117 nurses working in four different units (medical ward, surgical ward, intensive care unit, emergency room), and data from 351 shifts were used in the final analysis. Between-group differences in the main variables were analyzed using the t-test, the Mann–Whitney test, analysis of variance, or the Kruskal–Wallis test, as appropriate. The relationships were examined through multiple linear regression analysis.

**Results:**

The average number of steps and distance traveled were greatest for nurses working in the emergency room, followed by the intensive care unit, surgical ward, and medical ward (in descending order). Younger nurses and those with shorter unit experience tended to have the greatest number of steps and distance traveled.

**Conclusion:**

Using activity trackers, this study derived physical activity measures such as number of steps and distance traveled, enabling an objective examination of physical activity during shifts. Nurses’ level of physical activity differed depending on the type of nursing unit, nurses’ age, and unit experience. These results suggest the need for support programs that are specific to the job demands of specific nursing units.

**Supplementary Information:**

The online version contains supplementary material available at 10.1186/s12960-022-00768-3.

## Background

Nurses have a very complex work environment with a wide variety of job demands, encompassing physical, mental, psychological, and social aspects [1]. Physical demands are directly related to hospital nurses’ provision of direct patient care. Since nursing work should be performed rapidly and accurately [2], the physical demands are expected to be very high.

The primary physical activities nurses perform while on duty can be classified as standing [3], walking, running [3, 4], lifting objects, moving objects or devices and items, moving patients, changing patient positions, supporting patient ambulation, dragging wheelchairs, providing hygienic care to patients, and changing bed sheets [5, 6]. Walking and running are basic physical activities that are required in all nursing units and occur while going back and forth between the patient's bed and the nursing station [7]. A previous study that examined the workload and physical activity of nurses by measuring the number of steps and distance traveled during their work confirmed that during 12 h of work, nurses walked a number of steps corresponding to the daily step count of healthy adult men and women who were quite active [4]. Since that study only analyzed measurements made during working hours [4], which are limited in time and space, it is reasonable to predict that the number of steps per day of nurses will be significantly higher than that of adults of the same age.

In a study that analyzed the physical demands of nurses in relation to a more diverse range of activities, a significant difference was found in the degree of back pain of nurses as the intensity of physical activity increased [5]. It has also been reported that musculoskeletal disorders in nurses are related to occupational physical demands [8], and nurses who had more complex musculoskeletal pain with leg or foot disorders reported a higher intention to leave [9]. Additionally, a high quantity of job demands makes nurses feel unhealthy and increases their turnover intention [2]. Because it is reasonable to expect that an increased quantity of work would lead to increased physical activity, nurses' work performance and physical activity should be managed on the basis of more detailed analyses. Since the amount and intensity of physical activity at work are related to nurses’ health, especially musculoskeletal health, having musculoskeletal disorders might make it difficult for nurses to continue working. In addition, if nurses perform physically active tasks in an unhealthy state, fatigue could be accumulated and negatively affect patient safety [6].

One of the typical characteristics of nurse work is shift work, since nurses have a 24-h direct care presence [6]. Nurse’s shift work is an important factor because it is known to be closely associated with job performance [10], job satisfaction [11, 12] and health [12–14]. In previous studies analyzing physical activity among nurses working shifts, the intensity of physical activity differed across shifts, and there were also differences according to the sequence of shifts [6, 15]. These previous studies [6, 15] attempted to analyze the characteristics of nurses' shift work and physical activity using objective indicators, but it was not possible to investigate detailed aspects of the work content or intensity, such as nurse staffing or patients’ needs. In addition, most previous studies focused on comparing day and night shifts, without consideration of nurses’ 24-h, three-shift work nature [3, 6, 16, 17].

In addition, although relatively few studies have used objective indicators to quantify nurses’ physical activity at work, there has been an overall increase in studies using objective indicators within the last 10 years [6]. For example, studies using devices such as pedometers or activity trackers [4, 7, 15–21] and using observational methods [3] have been conducted to collect data from nurses in objective ways. However, many studies related to nurses’ job demands, including physical activity, have analyzed subjective measurements obtained using self-reporting questionnaires [1, 2, 5, 22–26]. Subjective perceptions are reliable when they are measured using a validated tool [27], but questionnaires are subject to limitations such as recall bias and social desirability bias [6]. Therefore, it is necessary to conduct research that can overcome these limitations by identifying differences or homogeneity between data sources by collecting both subjective data and objective data for a comparative analysis. Indicators of physical activity that could be commonly used in all nursing units, such as the number of steps and distance traveled, can be compared and analyzed according to nurses’ personal characteristics and the characteristics of nursing units. It will also be possible to calculate the time input according to nurses’ physical activity in the future and use this information to calculate the appropriate nursing workforce.

Therefore, in this study, an activity tracker was used to measure physical activity in terms of the number of steps and distance traveled for each shift, and nurses’ perceptions of physical demands were investigated at the same time. The aims of this study were to examine the relationship between nurses’ objective physical activity and their subjective perceptions, and to elucidate the differences in physical demands by shift (day, evening, and night) and factors associated with differences in physical activity.

## Methods

### Study design

This cross-sectional, descriptive study was conducted to examine nurses’ physical activity at work and related factors.

### Setting and participants

The subjects of this study were registered nurses working three shifts, and the survey was conducted at two tertiary hospitals located in the capital region of Korea. The two tertiary hospitals that participated in the study were selected to be similar in size and operational characteristics (private hospital, educational hospital, team nursing, etc.) to minimize variation between hospitals. In South Korea (hereafter Korea), hospitals are classified into hospitals (primary hospitals), general hospitals (secondary hospitals), and tertiary hospitals. A tertiary hospital must have at least 500 beds and sufficient staff, equipment, and facilities to treat seriously ill patients [28]. In addition, the Ministry of Health and Welfare designates tertiary hospitals through an assessment considering the composition of patients by disease group and the level of medical services. The two tertiary hospitals that participated in this study had 1179 and 765 beds as of the time of participation in the study, respectively (Additional file 1). Both of them were university hospitals involved in student education. Although there was a difference in bed size between the two hospitals, it was judged that the composition and severity of patients were similar, and accordingly nurses’ workloads were expected to be similar. In the two selected hospitals, the medical ward, surgical ward, intensive care unit, and emergency room, which are representative nursing units that perform three-shift work, were selected in consultation with the nursing department of each hospital, and nurses from a total of eight nursing units participated. The survey included all staff nurses who were dedicated to direct patient care in the nursing units; therefore, nursing managers were not included in the survey.

An attempt was made to obtain an even distribution of participants in terms of length of experience in each ward (less than one year, between one and three years, more than three years). The selection criteria also included possession of a personal smartphone, the ability to use an activity tracking application, and the possibility of working three different shifts during the data collection period. In addition to those who did not satisfy the above criteria, nurses who did not provide direct patient care were excluded from the study participation.

The required sample size for this study was calculated by setting an effect size of 0.15, a significance level of 0.05, and a power of 0.95 in the G*Power program. This yielded a result of 107. Considering a dropout rate of about 10%, a total of 120 nurses (15 from each of the eight wards) were recruited. During the process of data collection through the activity tracker and completion of a questionnaire, three people discontinued participation in the study, and data from 117 participants were included in the final analysis.

### Data collection

Subjects were recruited with the cooperation of the nursing department from two tertiary hospitals. Nurses from the four nursing units selected by each hospital were asked to participate in the study voluntarily. Data collection was conducted directly by the researchers for about two months from January 3, 2017 to March 15, 2017.

Four surveys were administered to subjects. The first survey investigated nurses’ general characteristics, workplace characteristics, and perceived physical demands through a questionnaire before wearing the activity tracker. In the second to fourth surveys, the number of steps and distance traveled were measured for each of the three shifts by wearing an activity tracker, and nurses were asked to fill out a questionnaire immediately after work on the day when measurements were made. The nurses wore an activity tracker according to their work schedules during a period of about a month and measured the number of steps and distance traveled during working hours. The measured data were recorded through a mobile phone application, and the nurses directly sent them to the researcher as a photo file. Although no direct requests were made related to the order of shifts (day, evening, and night), the participants were encouraged to avoid abnormal schedules or sudden changes in work schedules as much as possible. Therefore, data from 351 shifts were collected, corresponding to day, evening, and night shifts worked by each of the 117 participants.

### Measures

The initial questionnaire was used to gather general characteristics of the subjects, including gender, age, education level, unit experience, and type of nursing unit. To measure perceived physical demands, the nurses were asked to respond to the following question: “If you use 100 points as the standard for physical effort (energy) you put in during your day shift, please record the scores (0–100 points) of physical demands for your evening and night shifts.”

A smart band worn on the wrist was used as an activity tracker to measure the total number of steps and distance during shifts [29]. The smart band used in the survey was linked to a mobile phone application through Bluetooth, even if there was a certain distance from the mobile phone. The number of steps and distance traveled were recorded for each time period using the smart band’s accelerometer. A previous study confirmed that this smart band had an accuracy of 85% or more for measuring the number of steps, except when individuals walked with a cane [30]. To prevent differences or errors in measurement methods among participants, the researcher met the study subjects one-on-one and explained how to wear the smart band and how to use the mobile phone application. The participants were asked to transmit the number of steps and distance results measured immediately after their day, evening, and night shifts to the researcher as they were recorded in the application. The researcher reviewed the results and, if there was a possibility of error, discarded the data and asked participants to measure it again on the next shift to prevent the inclusion of erroneously measured data. Errors that actually occurred in measurements made using the activity tracker included situations where the step recording stopped as the link between the activity tracker and the mobile phone application was cut off while a nurse was working wearing a smart band, and where measurements were not performed due to a malfunction of the smart band device.

### Data analysis

The general characteristics of nurses were analyzed as frequency, percentage, mean, and standard deviation. Nurses’ total working hours, total number of steps, total distance traveled, and perceived physical demands were analyzed as mean and standard deviation values for each of the three shifts, and differences were compared using the Kruskal–Wallis test, followed by a post hoc test with the Bonferroni correction. Associations between nurses’ general characteristics and the total number of steps and total distance traveled during three shifts were tested by analysis of variance, the Kruskal–Wallis test, the t-test, or the Mann–Whitney test, followed by the Bonferroni post hoc test, as appropriate. The factors related to the total number of steps and distance traveled on the three shifts were analyzed through multiple linear regression. The average number of steps and distance traveled according to the three shift types (day, evening, and night) were used as dependent variables for multiple regression analysis. The independent variables, such as type of nursing unit, age, education level, and unit experience, were included in the multiple regression because they were statistically significant in the simple regression analysis, which was conducted before the multiple regression analysis.

### Ethical considerations

The study received institutional review board approval from Seoul National University (IRB No. 1701/001–002) before data collection was conducted. Nurses participated in the study on a voluntary basis and completed a written consent form prior to answering the survey questionnaires. In the consent form, the purpose of the research and confidentiality were presented. In particular, although the survey was conducted under the cooperation of the nursing department, it was stated that the responses of individual nurses were not disclosed at all and that confidentiality was thoroughly maintained. Participants were also informed that they could discontinue participation in the survey at any time without any disadvantage. Their personal information was managed in a master file separately from the datasets used for analysis.

## Results

The general characteristics of the study subjects are presented in Table 1. The subjects of this study were 117 nurses working three shifts. Most of them were women (94.0%) and the average age was 26.2 ± 3.3 years. Most of the participants had a bachelor's degree (83.8%). There was a relatively even distribution of participants across the medical ward (23.1%), surgical ward (25.6%), intensive care unit (25.6%), and emergency room (25.6%). The average unit experience was 2.9 ± 3.1 years.

**Table 1:** Demographic characteristics of participants (*N* = 117)

Characteristics	*M* ± SD	*n* (%)
Gender		
Female		110 (94.0)
Male		7 (6.0)
Age (year)	26.2 ± 3.3	
22–24		45 (38.5)
25–27		40 (34.2)
≥ 28		32 (27.4)
Education		
Associate degree		17 (14.5)
Bachelor’s degree		98 (83.8)
Master’s or higher		2 (1.7)
Unit type		
Medical ward		27 (23.1)
Surgical ward		30 (25.6)
Intensive care unit		30 (25.6)
Emergency room		30 (25.6)
Unit experience (year)	2.9 ± 3.1	
< 1		37 (31.6)
1 ≤ < 3		43 (36.8)
≥ 3		37 (31.6)

*M *mean, *SD* standard deviation

Nurses’ actual working hours including overtime, number of steps, distance traveled, and perceived physical demands when working in three shifts are presented in Table 2. Data from 351 shifts were analyzed, corresponding to three shifts each from the 117 participants. The average working hours of the nurses were 9.13 ± 1.07 h on the day shift, 9.09 ± 0.94 h on the evening shift, and 9.81 ± 1.25 h on the night shift. The difference in working hours was statistically significant among shifts (*p* < .001).

**Table 2:** Actual work hours, total number of steps, distance traveled, and perceived physical demands during work by shift (351 shifts from 117 nurses)

Variables	Shift	M ± SD	Min.	Max.	*H*	*p*	Bonferroni
Actual working hours including overtime	Day^a^ (*n* = 117)	9.13 ± 1.07	8.00	12.50	63.53	< .001	a,b < c
	Evening^b^ (*n* = 117)	9.09 ± 0.94	8.00	12.00			
	Night^c^ (*n* = 117)	9.81 ± 1.25	9.00	14.00			
	All	9.44 ± 0.73	8.00	14.00			
Total number of steps during the shift (1000 steps)	Day^a^ (*n* = 117)	9.78 ± 3.72	3.26	20.49	10.33	.006	a,b > c
	Evening^b^ (*n* = 117)	9.72 ± 4.07	2.91	22.22			
	Night^c^ (*n* = 117)	8.58 ± 4.01	2.64	19.66			
	All	9.36 ± 3.55	2.64	22.22			
Total distance traveled (1000 m)	Day^a^ (*n* = 117)	5.97 ± 2.32	1.30	12.60	8.29	.016	a,b > c
	Evening^b^ (*n* = 117)	6.07 ± 2.73	1.70	15.60			
	Night^c^ (*n* = 117)	5.32 ± 2.51	1.64	13.00			
	All	5.79 ± 2.20	1.30	15.60			
Perceived physical demands	Day^a^ (*n* = 117)	100			57.66	< .001	a,b > c
	Evening^b^ (*n* = 117)	96.75 ± 31.14	50.00	300.00			
	Night^c^ (*n* = 117)	90.09 ± 22.44	50.00	200.00			

^a, b,^ and ^c^ were indicated for group comparison of the Bonferroni test

The total number of steps during working hours was 9784 ± 3716 steps on the day shift, 9720 ± 4072 steps on the evening shift, and 8576 ± 4009 steps on the night shift; this difference was statistically significant (*p* = .006).

The distance traveled during working hours was 5971 ± 2323 m on the day shift, 6065 ± 2726 m on the evening shift, and 5321 ± 2512 m on the night shift. The shifts showed a statistically significant difference for this parameter (*p* = .016). The average distance traveled across all three shifts was 5786 ± 2197 m.

The physical demands for each shift perceived by nurses were relative scores for the evening and night shifts compared to a score of 100 for the day shift. The average score for the evening shift was 96.75 ± 31.14 points and that of the night shift was 90.09 ± 22.44 points. Perceived physical demands showed a statistically significant difference among the three shifts (*p* < .001).

The number of steps and distance traveled during three shifts were compared according to the general characteristics of the nurses, as presented in Table 3. The number of steps on all three shifts was highest in the emergency room (11,801 ± 4216), followed by the intensive care unit (10,277 ± 2872), medical ward (8081 ± 2097), and surgical ward (7153 ± 2587), and the difference was statistically significant (*p* < .001). Younger age showed a statistically significant association with a higher number of steps on all shifts (*p* < .001). Nurses with a bachelor's degree or higher had a higher number of steps on all three shifts, and this difference was significant for the night shift (*p* = .015) and all shifts (*p* = .049). Shorter unit experience was associated with a significantly higher number of steps on all three shifts (*p* < .001).

**Table 3:** Number of steps and distance traveled during the shift according to nurses’ general characteristics

Characteristics	Number of steps during the shift (1000 steps)				Distance traveled during the shift (1000 m)			
	Day (*n* = 117)	Evening (*n* = 117)	Night (*n* = 117)	All shifts (*N* = 351)	Day (*n* = 117)	Evening (*n* = 117)	Night (*n* = 117)	All shifts (*N* = 351)
	M ± SD				M ± SD			
Unit type								
Medical ward^a^	8.66 ± 2.53	8.51 ± 2.58	7.07 ± 2.68	8.08 ± 2.10	5.28 ± 1.56	5.18 ± 1.62	4.31 ± 1.71	4.92 ± 1.33
Surgical ward^b^	8.00 ± 3.08	7.14 ± 2.86	6.32 ± 2.91	7.15 ± 2.59	4.96 ± 1.97	4.42 ± 1.85	3.94 ± 1.84	4.44 ± 1.68
Intensive care unit^c^	10.97 ± 3.27	10.20 ± 2.94	9.66 ± 3.48	10.28 ± 2.87	6.91 ± 2.17	6.48 ± 1.95	6.09 ± 2.15	6.49 ± 1.86
Emergency room^d^	11.40 ± 4.55	12.91 ± 4.96	11.10 ± 4.70	11.80 ± 4.22	6.67 ± 2.81	8.10 ± 3.49	6.85 ± 2.94	7.20 ± 2.51
F/H/U(*p*)	16.8 (< .001)^†^	33.0 (< .001)^†^	28.4 (< .001)^†^	33.0 (< .001)^†^	15.5 (.001)^†^	32.6 (< .001)^†^	28.9 (< .001)^†^	33.4 (< .001)
Bonferroni	a, b < d, b < c	a, b, c < d, b < c	a, b < c, a, b < d		a, b < c, b < d	a, b c < d, b < c	a, b < c, d	
Age (year)								
22–24^a^	10.97 ± 3.80	10.60 ± 4.26	10.01 ± 3.77	10.86 ± 3.50	6.57 ± 2.39	7.28 ± 3.01	6.19 ± 2.40	6.68 ± 2.13
25–27^b^	9.94 ± 3.07	8.72 ± 3.34	8.31 ± 3.65	8.99 ± 2.87	6.10 ± 1.88	5.40 ± 2.15	5.18 ± 2.25	5.56 ± 1.79
≥ 28^c^	7.91 ± 3.69	8.33 ± 3.72	6.88 ± 4.14	7.71 ± 3.60	4.97 ± 2.45	5.19 ± 2.38	4.28 ± 2.62	4.81 ± 2.32
F/H/U(*p*)	14.7(< .001)^†^	17.7(< .001)^†^	16.8(< .001)^†^	19.2(< .001)^†^	12.0(.003)^†^	15.9(< .001)^†^	16.4(< .001)^†^	17.9(< .001)^†^
Bonferroni	a, b > c	a > b, c	a > c		a > c	a > b, c	a > c	
Education								
Associate degree	9.10 ± 3.50	8.55 ± 3.97	6.73 ± 3.61	8.13 ± 3.35	5.55 ± 2.09	5.15 ± 2.43	4.12 ± 2.17	4.94 ± 2.02
Bachelor’s degree or higher	9.90 ± 3.76	9.92 ± 4.07	8.89 ± 4.01	9.57 ± 3.55	6.04 ± 2.36	6.22 ± 2.75	5.53 ± 2.52	5.93 ± 2.20
F/H/U(*p*)	886.0 (.369)^††^	811.0 (.141)^††^	684.0 (.015)^††^	745.0 (.049)^††^	− 0.8 (.424)	783.0 (.092)^††^	687.5 (.016)^††^	726.0 (.035)^††^
Unit experience (year)								
< 1^a^	11.58 ± 3.78	11.74 ± 4.12	10.86 ± 3.93	11.39 ± 3.43	7.18 ± 2.36	7.32 ± 2.78	6.67 ± 2.41	7.05 ± 2.15
1 ≤ < 3^b^	10.19 ± 3.46	9.94 ± 4.15	8.81 ± 4.03	9.65 ± 3.46	6.04 ± 2.16	6.24 ± 2.85	5.53 ± 2.59	5.94 ± 2.09
≥ 3^c^	7.52 ± 2.72	7.45 ± 2.60	6.02 ± 2.31	6.99 ± 3.46	4.68 ± 1.78	4.61 ± 1.70	3.73 ± 1.48	4.34 ± 1.42
F/H/U(*p*)	24.0 (< .001)^†^	21.8 (< .001)^†^	29.8 (< .001)^†^	32.1 (< .001)^†^	22.6 (< .001)^†^	20.3 (< .001)^†^	29.3 (< .001)^†^	31.7 (< .001)^†^
Bonferroni	a, b > c	a, b > c	a > b, c, b > c		a < b, c, b > c	a, b > c	a, b > c	

^†^Kruskal–Wallis test, ^††^Mann–Whitney test, *M* = mean, SD = standard deviation

^a, b, c,^ and ^d^ were indicated for group comparison of the Bonferroni test

Significant differences in distance traveled were found by unit for the day (*p* = .001), evening (*p* < .001), and night (*p* < .001) shifts. On the day shift, the longest distance traveled was found for the intensive care unit (6911 ± 2172), followed in descending order by the emergency room (6667 ± 2807), medical ward (5278 ± 1557), and surgical ward (4957 ± 1970). However, on the evening and night shifts, the longest distance traveled was found in the emergency room, followed in order by the intensive care unit, medical ward, and surgical ward. Older age was significantly associated with distance traveled on the day (*p* = .003), evening (*p* < .001), and night (*p* < .001) shifts. Educational level only showed a significant difference on the night shift (*p* = .016), although nurses with a bachelor's degree or higher traveled a longer distance on all shifts. Shorter unit experience was significantly associated with a longer distance traveled on all three shifts (*p* < .001 for all).

Multiple linear regression was performed to confirm the factors related to the number of steps per shift, and the results are as follows (Table 4). The intensive care unit (β = 2.03, *p* = .025) and the emergency room (β = 2.41, *p* = .007) showed significant positive associations compared to the medical ward on the day shift. The emergency room showed a significant association (β = 4.10, *p* < .001) on the evening shift, while significant positive relationships were found for the intensive care unit (β = 1.95, *p* = .034) and emergency room (β = 3.78, *p* < .001) on the night shift. No factors showed a statistically significant effect according to education level on any of the three shifts. Significant negative associations were found with nurses’ unit experience on the day (β = − 0.05, *p* = .004), evening (β = − 0.04, *p* = .022), and night (β = − 0.06, *p* = .001) shifts.

**Table 4:** Relationships between steps during three shifts and characteristics of nurses: regression coefficients (*N* = 117)

Characteristics	Number of steps during the shift								
	Day			Evening			Night		
	Coefficient (95% CI)	SE	*p*	Coefficient (95% CI)	SE	*p*	Coefficient (95% CI)	SE	*p*
Unit type (vs. medical ward)									
Surgical ward	− 0.07 (− 1.82, 1.68)	0.88	.938	− 0.91 (− 2.71, 0.88)	0.91	.317	− 0.16 (− 1.94, 1.62)	0.90	.860
Intensive care unit	2.03 (0.26, 3.80)	0.89	.025	1.26 (− 0.56, 3.08)	0.92	.172	1.95 (0.15, 3.75)	0.91	.034
Emergency room	2.41 (0.67, 4.15)	0.88	.007	4.10 (2.32, 5.89)	0.90	< .001	3.78 (2.01, 5.55)	0.89	< .001
Age	0.24 (− 0.15, 0.64)	0.20	.225	0.20 (− 0.21, 0.60)	0.20	.337	0.38 (− 0.02, 0.78)	0.20	.061
Educational level (vs. associate degree)	− 0.30 (− 1.97, 1.38)	0.84	.727	0.81 (− 0.91, 2.52)	0.87	.354	1.14 (− 0.57, 2.84)	0.86	.188
Unit experience	− 0.05 (− 0.09, − 0.02)	17.44	.004	− 0.04 (− 0.08, − 0.01)	0.18	.022	− 0.06 (− 0.09, − 0.02)	0.02	.001

*SE* standard error, *CI *confidence interval

The factors related to the distance traveled on shifts were also confirmed through multiple linear regression, and the results are presented in Table 5. The intensive care unit (β = 1.44, *p *= .012) and emergency room (β = 1.22, *p *= .031) showed significant positive relationships on the day shift, the emergency room (β = 2.74, *p *< .001) demonstrated a positive relationship on the evening shift, and significant positive associations were found for the intensive care unit (β = 1.41, *p *= .016) and emergency room (β = 2.34, *p *< .001) on the night shift. No factors showed a statistically significant association according to education level across the three shifts. Significant negative relationships were found with nurses’ unit experience on the day (β = − 0.04, *p *= .002), evening (β = − 0.03, *p *= .024), and night (β = − 0.04, p = .002) shifts.

**Table 5:** Relationships between distance traveled during three shifts and characteristics of nurses: regression coefficients (*N* = 117)

Characteristics	Distance traveled during the shift								
	Day			Evening			Night		
	Coefficient (95% CI)	SE	*p*	Coefficient (95% CI)	SE	*p*	Coefficient (95% CI)	SE	*p*
Unit type (vs. medical ward)									
Surgical ward	0.08 (− 11.03, 1.18)	0.56	.890	− 0.46 (− 1.68, 0.76)	0.62	.456	− 0.002 (− 1.12, 1.12)	0.57	.997
Intensive care unit	1.44 (0.32, 2.56)	0.56	.012	0.99 (− 0.24, 2.23)	0.62	.113	1.41 (0.27, 2.54)	0.57	.016
Emergency room	1.22 (0.12, 2.31)	0.55	.031	2.74 (1.53, 3.95)	0.61	< .001	2.34 (1.27, 3.50)	0.56	< .001
Age	0.20 (− 0.05, 0.45)	0.13	.108	0.14 (− 0.13, 0.42)	0.14	.305	0.23 (− 0.02, 0.48)	0.13	.073
Educational level (vs. associate degree)	− 0.30 (− 1.36, 0.76)	0.539	.576	0.65 (− 0.52, 1.81)	0.59	.275	0.63 (− 0.44, 1.70)	0.54	.246
Unit experience	− 0.04 (− 0.06, − 0.01)	0.01	.002	− 0.03 (− 0.05, − 0.004)	0.01	.024	− 0.04 (− 0.06, − 0.14)	0.01	.002

*SE* standard error, *CI* confidence interval

## Discussion

This study investigated nurses’ physical demands at work using both objective and subjective measurement methods. Nurses’ number of steps and distance traveled on each shift were measured objectively using an activity tracker, and the subjective perceptions of nurses' physical demands for each shift were surveyed through questionnaires. Measurements of the number of steps and the distance traveled for three shifts were made for all nurses who participated in this study. Accordingly, it was possible to include data from the evening shift in the comparative analysis in this study, unlike previous studies [4, 16, 17]. In order to overcome the limitations of a previous study that used a pedometer [4], physical activity was measured using a reliable smart band [30] that was light enough for nurses to wear while working.

The average number of steps that nurses walked while working an average of 9.4 h per shift was 9360 steps, and the distance traveled was 5.79 km, which is consistent with the results of a study [4] that measured the number of steps and walking distance for nurses. The previous study suggested that the nurses’ workflow was related to their workload and found significant differences between day and night shifts and depending on the number of patients nurses were responsible for [4]. Another previous study measured the walking distance of nurses during 10 h of work and showed a shorter measured value than the walking distance measured in this study [7]. However, the present study is consistent with earlier research in that it found a longer walking distance on the day shift than on the night shift [4, 7, 16]. In a previous study that measured physical activity in the general public, the average number of steps per day was 8385 steps for women in general and 8875 steps for women aged 20 to 39 [31]. In another similar study, an average of 7500 to 9999 steps per day was classified as somewhat active, 10,000 to 12,499 steps as active, and 12,500 or more steps as very active; in that study, the average number of steps for women per day was 6486 [32], indicating that the average physical activity of nurses was higher compared to the general population.

This study found that nurses worked long hours on night shifts and walked more than 8000 steps. The number of steps and the distance traveled during the night shift were lower than those of the day and evening shifts despite the significantly longer working hours. A previous study found that the average number of steps that nurses walked during the night shift was about 4000, whereas the number of steps during the day was more than 8000 [16]. In comparison, the number of steps walked during the night shift in this study was substantially higher. Even when compared with a study of shift workers in other occupations [33], nurses’ physical activity during the night shift was found to be higher. A comprehensive comparison of the results of the present study to those of previous studies [16, 33] showed that nurses' night shifts required considerable physical activity, as perceived by nurses themselves.

In this study, the highest number of steps was found for the day shift, followed in descending order by the evening and night shifts. In general, since treatment and surgery are performed during the daytime, it can be expected that nurses will be more physically active. However, these results were not consistent for all nursing units. Unlike other nursing units, the emergency room had the highest number of steps on the evening shift, followed in descending order by the day and night shifts, and by the night and day shifts for distance traveled. The emergency room involves a substantial amount of unpredictable work rather than routine work; therefore, it is reasonable to assume that nurses’ physical workload will increase when unpredictable patients more frequently visit the emergency room. Of note, while both the number of steps and distance traveled were higher on the evening shift, contradictory trends were found between the day and night shifts for these parameters, which means that a high number of steps do not always correspond to a long distance. Depending on the details of the job, nurses may have to take many steps in a narrow space, or they may have to walk a long distance to carry out their tasks with a wider stride. To further clarify the difference between the number of steps and the distance traveled, further research should investigate the association between nurses’ specific work tasks and physical activity.

The objectively measured results for the number of steps and distance traveled using an activity tracker showed the same pattern as the physical activity scores perceived by nurses. When the score for physical activity during the day shift was set to 100 points and nurses were asked to score the physical activity of the other shifts relative to the day shift, the scores for the evening and night shifts were 96.75 and 90.09 points, respectively. The corresponding ratios using the actual measured number of steps were 99.35 for the evening shift and 87.65 for the night shift, showing close alignment between the subjective and objective measurements. A previous study that measured and compared the physical activity of nurses with objective and subjective indicators found that while nurses overestimated their walking time, their standing time was underestimated [19]. It was reported that the time nurses worked while standing was about four times higher than the time that they actually walked, and that when providing nursing care beside of the patients, they were more likely to perceive themselves as walking even when they were only taking a few steps while standing [19]. Even considering that nurses are likely to confuse walking with standing during work, it is nonetheless clear that nurses’ subjective perceptions of their physical activity during work are relatively accurate.

In the present study, the emergency room had the highest number of steps and distance traveled compared to other nursing units, followed by the intensive care unit. These units had significantly higher values than were measured in the general ward. Although it might be reasonable to predict that nurses in general wards, who care for more patients and are required to move throughout a larger area, the present study instead found that nurses in special care units with a limited space walked much more than those in the general wards. Because nurses’ jobs require a variety of physical activities that depend on the occupational setting [5], it is necessary to investigate and compare the types and intensities of physical activities to reveal differences in physical activity and nursing performance according to nursing department and nursing content. However, this study only investigated and compared the physical activities required in general. Therefore, additional research is needed for a more specific analysis of physical activities in specialized wards such as emergency rooms and intensive care units.

In addition, it was found that the number of steps and distance traveled was higher among younger nurses and those with less work experience. The average number of steps for nurses with less than one year of unit experience was 11,000 or more, whereas it was only about 7000 steps for those with three years or more of experience, meaning that the least experienced nurses walked about 40% more than nurses with three or more years of experience. This discrepancy can be accounted for by differences in competency and roles according to career years. For example, nurses with less than one year of experience are more likely to spend more time in direct nursing care than nurses with longer experience, and may need to walk back and forth between the nursing station and patient rooms more frequently if they miss necessary items due to a lack of experience. Multiple regression analysis in the present study also confirmed that the number of steps and distance traveled were significantly higher in the emergency room and intensive care unit than in the medical ward on all three shifts, as well as in nurses with shorter unit experience. In the comparative analysis between groups, participants with a higher education level showed significantly higher number of steps and distance traveled. However, in the multiple regression analysis, the education level did not significantly affect nurses’ steps and distance traveled. Collectively, these findings indicate that nurses’ physical activity, such as the number of steps and distance traveled, is most directly affected by the specificity of the nursing unit and the nurse's unit experience.

Previous studies have examined the relationships between nurses’ job demands and health [2, 8, 10, 11, 13, 14, 34, 35]. Consistent reports from around the world have found that nurses suffer from musculoskeletal problems related to nursing work [5, 8, 19, 34, 36]. Since musculoskeletal problems are particularly likely to be linked to physical activity, it is necessary to conduct further analyses according to the characteristics and intensity of various physical activities during nurses’ work. A detailed list of physical activities should be compared with consideration of the nursing unit, nursing content, and individual characteristics in order to predict and manage the health risks related to physical activity. Scales such as the Therapeutic Intervention Scoring System (TISS-28 Scale) and Nursing Activity Score (NAS) have been developed to measure nurses' activities, mainly by quantifying nursing intensity from the patient's side [37]. Accordingly, those scales have limitations insofar as they can only measure major nursing activities related to the patient's condition. Therefore, through development of a scale that can comprehensively measure nurses’ actual work activities, physical demands and consequent physical and mental fatigue would enable a more accurate analysis. For example, a time-motion study could inform the development of a scale that would measure the time taken according to a list of all nurses' activities and score them by applying the intensity according to the patient's condition. Such a scale could be used to measure activity and fatigue for individual nurses, thereby providing data to understand the health status of nurses and manage their health. Through such initiatives, it would be possible to develop a health management program to reduce accumulated fatigue in body parts that are frequently used at work. This information could also be used for nursing managers to designate a working pattern or shift type suitable for nurses according to their health conditions and to supplement human resources in advance or to allocate them efficiently in the field.

There are several limitations of this study. First, although seven male nurses were included in the study, gender-related differences were not included in the analysis. Second, cautious interpretation is needed for the results of this study because the nurses who participated in the study were relatively young and the survey period was short. Third, this study was conducted at two tertiary hospitals, limiting the generalizability of the results. Because tertiary hospitals are substantially different from other hospital types in the number of beds, staff, facility size, and patient severity, further research is needed that would compare nurses’ physical demands according to the hospital category. Furthermore, even in the same nursing unit, there may be differences in nurses’ number of steps depending on the size of the unit and the number of nursing staff (Additional file 2). The results of this study should be interpreted with caution because all these points were not considered in detail and the classification was made according to the nursing unit. Fourth, although the smart band used in this study was easy to wear and light enough not to influence nurses’ ability to move, there is a possibility that wearing an activity tracker might affect nurses’ intention to walk more, thereby leading to measurements that suggest inaccurately intense physical demands in some areas. Fifth, because nurses from various nursing units participated, the number of participants in each group may not be sufficient to compare the results of nurse groups between units. In order to analyze differences between units in the future, additional studies with more subjects are required.

## Conclusions

In this study, physical activity was measured objectively as the number of steps and distance traveled, using an activity tracker, as has become common in real-life contexts in recent years. The number of steps and distance traveled showed differences according to the nursing unit, nurses’ age, and nurses’ unit experience throughout the three shifts. Based on these results, we suggest that nurses’ work-related physical activities should be predicted and managed with appropriate consideration of the work characteristics of nursing units, nurses’ age, and nurses’ career experience.

Follow-up studies using activity trackers can objectively measure and evaluate physical indicators such as changes in heart rate and the quantity and quality of sleep while working as a nurse would help to articulate the relationship between nurses' physical activity and health more accurately. In addition, it is expected that medical institutions can use activity trackers to collect and analyze the physical activity data of nurses in real time on a long-term basis, which could be used in decision-making regarding placement and supplementation of the nursing workforce.

## Supplementary Information

**Additional file 1.**  General characteristics of the participating hospitals.

**Additional file 2.**  General characteristics of the participating units from two hospitals.

## Data Availability

The data presented in this study are available on request from the corresponding author. The data are not publicly available due to privacy or ethical restrictions.

## References

[1] Scheepers RA, Smeulders IM, van den Broek T (2021). The impact of an additional nurse assistant during evening shifts on nurses’ perceptions of job demands, job resources and well-being. J Adv Nurs.

[2] Cho SH, Park M, Jeon SH, Chang HE, Hong HJ (2014). Average hospital length of stay, nurses’ work demands, and their health and job outcomes. J Nurs Scholarsh.

[3] Yu F, Narayanan A, Mackay L, Ward K, King A, Smith M (2020). Describing objectively measured intensive care nurses' physical work activity behavioural patterns during a 12-hr shift. J Clin Nurs.

[4] Welton JM, Decker M, Adam J, Zone-Smith L (2006). How far do nurses walk?. Medsurg Nurs.

[5] Jung K, Suh S (2013). Relationships among nursing activities, the use of body mechanics, and job stress in nurses with low back pain. J Muscle Joint Health.

[6] Chappel SE, Verswijveren SJ, Aisbett B, Considine J, Ridgers ND (2017). Nurses’ occupational physical activity levels: a systematic review. Int J Nurs Stud.

[7] Hendrich A, Chow M, Skierczynski BA, Lu Z (2008). A 36-hospital time and motion study: how do medical-surgical nurses spend their time?. Per J..

[8] Trinkoff AM, Le R, Geiger-Brown J, Lipscomb J, Lang G (2006). Longitudinal relationship of work hours, mandatory overtime, and on-call to musculoskeletal problems in nurses. Am J Ind Med.

[9] Ki J, Ryu J, Baek J, Huh I, Choi-Kwon S (2020). Association between health problems and turnover intention in shift work nurses: health problem clustering. Int J Environ Res Public Health.

[10] Dall’Ora C, Ball J, Recio-Saucedo A, Griffiths P (2016). Characteristics of shift work and their impact on employee performance and wellbeing: a literature review. Int J Nurs Stud.

[11] Josten EJC, Ng-A-Tham JE, Thierry H (2003). The effects of extended workdays on fatigue, health, performance and satisfaction in nursing. J Adv Nurs.

[12] Sagherian K, Clinton ME, Abu-Saad Huijer H, Geiger-Brown J (2017). Fatigue, work schedules, and perceived performance in bedside care nurses. Workplace Health Saf.

[13] Hansen AB, Stayner L, Hansen J, Andersen ZJ (2016). Night shift work and incidence of diabetes in the Danish Nurse Cohort. Occup Environ Med.

[14] Min A, Kang M, Hong HC (2021). Sickness presenteeism in shift and non-shift nurses: using the fifth Korean working conditions survey. Int J Environ Res Public Health.

[15] Chappel SE, Aisbett B, Considine J, Ridgers ND (2020). Emergency nurses' activity levels across rotating shifts. Australas Emerg Care.

[16] Kwiecień-Jaguś K, Mędrzycka-Dąbrowska W, Czyż-Szypenbeil K, Lewandowska K, Ozga D (2019). The use of a pedometer to measure the physical activity during 12-hour shift of ICU and nurse anaesthetists in Poland. Intensive Crit Care Nurs.

[17] Benzo R, Farag A, Whitaker KM, Xiao Q, Carr LJ (2021). A comparison of occupational physical activity and sedentary behavior patterns of nurses working 12-h day and night shifts. Int J Nurs Stud Adv.

[18] Donahue L (2009). A pod design for nursing assignments: eliminating unnecessary steps and increasing patient satisfaction by reconfiguring care assignments. Am J Nurs.

[19] Li J, Sommerich C, Lavender S, Chipps E, Stasny E, editors. Subjective and objective estimation of physical activities on the lower extremities for inpatient staff nurses and their lower extremity musculoskeletal discomfort. Proc Hum Factors Ergon Soc Annu Meet. 2017;61(1): 1017–21. 10.1177/1541931213601737.

[20] Jirathananuwat A, Pongpirul K (2017). Physical activity of nurse clinical practitioners and managers. J Phys Act Health.

[21] Van Poel E, Ketels M, Clays E (2020). The association between occupational physical activity, psychosocial factors and perceived work ability among nurses. J Nurs Manag.

[22] Spence K, Tarnow-Mordi W, Duncan G, Jayasuryia N, Elliott J, King J (2006). Measuring nursing workload in neonatal intensive care. J Nurs Manag.

[23] MacPhee M, Dahinten VS, Havaei F (2017). The impact of heavy perceived nurse workloads on patient and nurse outcomes. Adm Sci.

[24] Mc Carthy VJC, Wills T, Crowley S (2018). Nurses, age, job demands and physical activity at work and at leisure: a cross-sectional study. Appl Nurs Res.

[25] Laschinger HKS, Grau AL, Finegan J, Wilk P (2012). Predictors of new graduate nurses’ workplace well-being: testing the job demands–resources model. Health Care Manage Rev.

[26] Salyer J (1995). Environmental turbulence: impact on nurse performance. J Nurs Adm.

[27] Polit DF (2015). Assessing measurement in health: beyond reliability and validity. Int J Nurs Stud.

[28] Korean Ministry of Health & Welfare. Public health policies: Health organization policy. http://www.mohw.go.kr/react/policy/index.jsp?PAR_MENU_ID=06&MENU_ID=06290304&PAGE=4&topTitle= (2022). Accessed 25 Aug 2022.

[29] Xiaomi. Mi Band. 2017. Available from http://www.mi.com/en/miband.

[30] Lin F, Wang A, Song C, Xu W, Li Z, Li Q. A comparative study of smart insole on real-world step count. IEEE Signal Process Med Biol Symp. 2015:1–6. 10.1109/SPMB.2015.7405425.

[31] Colley RC, Garriguet D, Janssen I, Craig CL, Clarke J, Tremblay MS (2011). Physical activity of Canadian adults: accelerometer results from the 2007 to 2009 Canadian Health Measures Survey. Health Rep.

[32] Tudor-Locke C, Brashear MM, Johnson WD, Katzmarzyk PT (2010). Accelerometer profiles of physical activity and inactivity in normal weight, overweight, and obese US men and women. Int J Behav Nutr Phys Act.

[33] Kolbe-Alexander TL, Gomersall S, Clark B, Torquati L, Pavey T, Brown WJ (2019). A hard day’s night: time use in shift workers. BMC Public Health.

[34] Gershon RR, Stone PW, Zeltser M, Faucett J, Macdavitt K, Chou S-S (2007). Organizational climate and nurse health outcomes in the United States: a systematic review. Ind Health.

[35] Jung YJ, Kang SW (2017). Differences in sleep, fatigue, and neurocognitive function between shift nurses and non-shift nurses. Korean J Adult Nurs.

[36] Jun K-J (2009). Occupational diseases and injuries among Korean nurses. Korean J Occup Health Nurs.

[37] Kwiecień K, Wujtewicz M, Mędrzycka-Dąbrowska W (2012). Selected methods of measuring workload among intensive care nursing staff. Int J Occup Med Environ Health.

