# Supplementary material for: Nurses’ steps, distance traveled, and perceived physical demands in a three-shift schedule
Source: Hum Resour Health. 2022 Oct 8;20:72. doi: 10.1186/s12960-022-00768-3 (PMC9548108; doi:10.1186/s12960-022-00768-3)
Supplement: Supplementary file 1 — Additional file 1. General characteristics of the participating hospitals. [file 12960_2022_768_MOESM1_ESM.docx]

**Additional File 1: General characteristics of the participating hospitals**

|  | Hospital | |
| --- | --- | --- |
| Characteristics | A | B |
| Hospital type | Tertiary (education) | Tertiary (education) |
| Hospital size | Large | Medium |
| Location | Seoul | Gyenggi-do |
| Number of beds | 1,179 | 765 |
| Number of participating nurses | 57 | 60 |
