# Supplementary material for: Nurses’ steps, distance traveled, and perceived physical demands in a three-shift schedule
Source: Hum Resour Health. 2022 Oct 8;20:72. doi: 10.1186/s12960-022-00768-3 (PMC9548108; doi:10.1186/s12960-022-00768-3)
Supplement: Supplementary file 2 — Additional file 2. General characteristics of the participating units from two hospitals. [file 12960_2022_768_MOESM2_ESM.docx]

**Additional File 2: General characteristics of the participating units from two hospitals**

|  | Type of nursing unit | Number of beds | Nurse to patient ratio (per shift) | Total number of nurses | Number of participants |
| --- | --- | --- | --- | --- | --- |
| Hospital A | Medical ward | 38 | 1:9.6 | 17 | 12 |
|  | Surgical ward | 55 | 1:12.1 | 21 | 15 |
|  | Mixed ICU | 36 | 1:2.5 | 59 | 15 |
|  | ER | 42 | 1:16.9 | 48 | 15 |
| Hospital B | Medical ward | 49 | 1:9.4 | 22 | 15 |
|  | Surgical ward | 49 | 1:8.7 | 20 | 15 |
|  | Surgical ICU | 18 | 1:3.2 | 29 | 15 |
|  | ER | 31 | 1:19.3 | 34 | 15 |

ICU=intensive care unit, ER=emergency room.
